# Laser fragmentation of MoS_2_ in ethanol: MoO_3−_*_x_* coatings, oxygen vacancies, and photoluminescence with NIR heating

**DOI:** 10.3762/bjnano.17.95

**Published:** 2026-10-08

**Authors:** José Guillermo Castillo-Lara, Miguel Ángel Camacho-López, Liliana Aranda-Lara, Marco Antonio Camacho-López, Jazmín Fuentes-Becerril, Keila Isaac-Olivé, Nallely Patricia Jiménez-Mancilla, Santiago Camacho-López, Luis Escobar-Alarcón, Noé Zamora-Romero

**Affiliations:** 1 Laboratorio de Fotomedicina, Biofotónica y Espectroscopia Láser de Pulsos Ultracortos, Facultad de Medicina, Universidad Autónoma del Estado de México, Jesús Carranza y Paseo Tollocan s/n, Toluca, 50180, México; 2 Laboratorio de Investigación en Teranóstica, Facultad de Medicina, Universidad Autónoma del Estado de México, Jesús Carranza y Paseo Tollocan s/n, Toluca, 50180, México; 3 Laboratorio de Investigación y Desarrollo de Materiales Avanzados, Facultad de Química, Universidad Autónoma del Estado de México, Campus Rosedal, Km 14.5 Carretera Toluca-Atlacomulco, Toluca, 50925, México; 4 Cátedras SECIHTI, Instituto Nacional de Investigaciones Nucleares, Carretera México-Toluca s/n, 52750, México; 5 Centro de Investigación Científica y de Educación Superior de Ensenada, Carretera Ensenada-Tijuana No. 3918 Ensenada, 22860, Baja California, México; 6 Laboratorio de Investigación en Nanomateriales Avanzados, Instituto Nacional de Investigaciones Nucleares, Carretera México-Toluca s/n, 52750, México

**Keywords:** core–shell nanostructures, fullerene-like MoS_2_, laser ablation in liquids, oxygen vacancies, photoluminescence

## Abstract

Two-dimensional transition metal dichalcogenides such as MoS_2_ are attractive optical nanomaterials because they can exhibit photoluminescence and light-to-heat conversion within the same colloidal system. Here, we synthesize surfactant-free Mo-based nanocolloids by picosecond pulsed laser ablation of a bulk MoS_2_ target in ethanol followed by laser-induced colloidal fragmentation. HRTEM shows that the ablation step initially yields 2H-MoS_2_ nanosheets with an interplanar spacing of 0.274 nm (assigned to the (100) planes), while subsequent fragmentation produces 12.3 ± 1.7 nm fullerene-like core–shell nanoparticles comprising a curved MoS_2_ core and an amorphous/poorly crystalline molybdenum oxide shell. Raman spectroscopy provides evidence consistent with the coexistence of 2H-MoS_2_ and sub-stoichiometric molybdenum oxide (MoO_3−_*_x_*). UV–vis spectra analyzed via Tauc plots yield an apparent optical bandgap of *E*_g_ ≈ 1.94 eV, suggesting size/curvature-related modifications of the electronic structure. Under 260 nm excitation, the nanocolloids display intense blue–violet photoluminescence with bands centered at 401, 425, and 452 nm. The emission is consistent with contributions from confined/curved MoS_2_ domains together with defect–related states associated with oxygen-deficient MoO_3−_*_x_*. Finally, irradiation with a 270 W near-infrared lamp produces a temperature rise of ~11 °C in 10 min for the nanocolloids, compared with ~6 °C for ethanol alone, indicating an enhanced photothermal heating response. These results highlight picosecond laser processing in liquids as a straightforward route to MoS_2_/MoO_3−_*_x_* fullerene-like nanostructures in which heating is obtained under broadband, spatially distributed irradiation rather than focused laser excitation, a configuration of interest for photothermal applications, where the local power densities delivered by focused sources are a recognised constraint.

## Introduction

Photoluminescence arising from crystalline defects in transition metal oxides provides a versatile route to engineer optically active nanomaterials. In sub-stoichiometric molybdenum oxide (MoO_3−_*_x_*), oxygen deficiency (*x* > 0) is associated with oxygen anion vacancies (V_o_) that introduce electronic states within the bandgap, enabling visible emission at room temperature. Because these defect states are sensitive to the local chemical environment, their density can influence both the intensity and the spectral position of the photoluminescence (PL) signal [1]. The formation of V_o_ in MoO_3−_*_x_* is commonly linked to partial reduction of Mo^6+^ to Mo^5+^ and the accompanying polaronic/intervalence states, which can broaden optical absorption toward the visible and support radiative transitions typically reported in the 420–550 nm range [2–3].

In MoS_2_/MoO_3−_*_x_* heterostructures, an oxygen-deficient oxide layer can contribute with additional defect-related emissive states and may facilitate interfacial charge transfer, leading to visible and excitation-dependent PL responses [4]. Reported MoS_2_/MoO_3−_*_x_* heterojunctions therefore motivate the construction of mixed sulfide/oxide nanosystems as optical transducers whose emission can reflect defect chemistry and interfacial electronic interactions. In the present work, ultraviolet excitation at 260 nm produces a multiband PL response that is discussed in the context of MoS_2_ confinement/curvature and oxygen-deficient molybdenum oxide states.

Beyond emission, MoS_2_-based nanostructures have also been explored for light-to-heat conversion under near-infrared (NIR) irradiation [4–7]. Accordingly, when MoS_2_ is integrated with an oxide shell, the resulting colloids may combine defect-related photoluminescence with an enhanced NIR-heating response. These considerations underscore the interest in surfactant-free MoS_2_/MoO_3−_*_x_* colloids that integrate optical emission with NIR lamp heating while enabling systematic correlations between structure, defect chemistry, and optical behaviour. Such heating is commonly evaluated using focused laser sources, whose local power densities at the irradiated surface are a recognised constraint in photothermal applications [8]; broadband lamp irradiation, employed here, provides an alternative configuration in which the incident power is spatially distributed over the sample.

Fullerene-like MoS_2_ nanostructures and MoS_2_/molybdenum oxide heterostructures have been obtained through several alternative routes. Closed-cage MoS_2_ nanoparticles were first reported from high-temperature gas-phase sulfidization of molybdenum oxide precursors [9–10], a route later extended to bulk synthesis from the respective trioxides [11] and refined through mechanistic studies of gas-phase growth [12]; related approaches include the reaction of solid precursors with sulfur in the presence of a hydrogen-releasing agent [13] and ablation under concentrated sunlight [14]. The oxygen-deficient oxide component, in turn, is most often prepared separately: Sub-stoichiometric molybdenum oxide with a controlled vacancy density is commonly obtained by hydrothermal or solvothermal treatment, in which the vacancy content is tuned through the amount of oxidizing agent or the reaction temperature [15–16], or an oxide layer is grown directly on MoS_2_ by controlled surface oxidation using O_2_ plasma, remote plasma exposure or thermal annealing [17–19].

These routes yield well-defined structures, but they generally operate at elevated temperatures, require additional reagents or reducing agents, and introduce the oxide as a post-treatment applied to a previously synthesized sulfide. By contrast, the two-step picosecond route employed here operates at ambient pressure and requires neither surfactants, capping agents, nor chemical reducing or oxidizing agents, and the curvature of the sulfide core and the formation of the oxygen-deficient oxide coating arise within the same processing step. Composition and morphology are thus governed by purely physical parameters (pulse duration, fluence, and irradiation time) at the cost of a broader size distribution than that typically achieved by wet-chemical routes.

In this context, MoS_2_-based colloids were produced in ethanol by picosecond pulsed laser ablation of a high-purity MoS_2_ target, followed by a laser-induced colloidal fragmentation step aimed at reshaping and downsizing the as-generated nanostructures. In the second stage, the solid target was removed and laser pulses were focused directly into the previously obtained suspension, a configuration known to promote fragmentation, re-dispersion, and structural rearrangement of colloidal nanomaterials [20–21]. Under such fragmentation conditions, transient high-pressure and high-temperature environments associated with cavitation events can drive curvature and closure of layered MoS_2_ nanosheet fragments, providing a plausible pathway for converting nanosheets into fullerene-like structures. In parallel, laser processing in ethanol may lead to solvent-derived reactive species that can promote partial surface oxidation, potentially yielding oxygen-deficient molybdenum oxide (MoO_3−_*_x_*) coatings. In the present work, HRTEM and Raman features are discussed as evidence consistent with the formation of fullerene-like MoS_2_ cores accompanied by sub-stoichiometric molybdenum oxide on the particle surface, where oxygen vacancies are expected to play a role in the observed photoluminescence [22–24].

The visible photoluminescence observed in the resulting nanocolloids is consistent with the combined contribution of (i) size/curvature-related effects associated with the nanosheet-to-fullerene-like transformation and (ii) defect-related states expected for oxygen-deficient molybdenum oxide (MoO_3−_*_x_*) coatings. Accordingly, the experimental observations support that picosecond laser processing in ethanol can generate surfactant-free MoS_2_-based nanostructures in which morphology and defect chemistry are modified without the use of dopants or additional chemical reducing/oxidizing agents. In this sense, laser ablation in liquids followed by re-irradiation/fragmentation provides a clean physical route to hybrid MoS_2_/MoO_3−_*_x_* colloids with tunable optical response.

Building on this framework, the present work reports the synthesis of MoS_2_/MoO_3−_*_x_* fullerene-like core–shell nanoparticles by laser ablation and colloidal fragmentation in ethanol, together with structural and spectroscopic characterization of the nanocolloids. The discussion emphasizes correlations between morphology (nanosheets versus fullerene-like particles), Raman/TEM evidence for sub-stoichiometric oxide formation, and the observed multiband photoluminescence under 260 nm excitation. In addition, a temperature-rise experiment under irradiation with a commercial NIR lamp is included to evaluate their NIR-heating response under simple, reproducible conditions. Overall, the study establishes a materials-processing and structure–property basis for future investigations where these surfactant-free colloids could be explored as optical transducers and light-to-heat nanoheaters in application-specific settings.

## Results and Discussion

**Figure 1:**
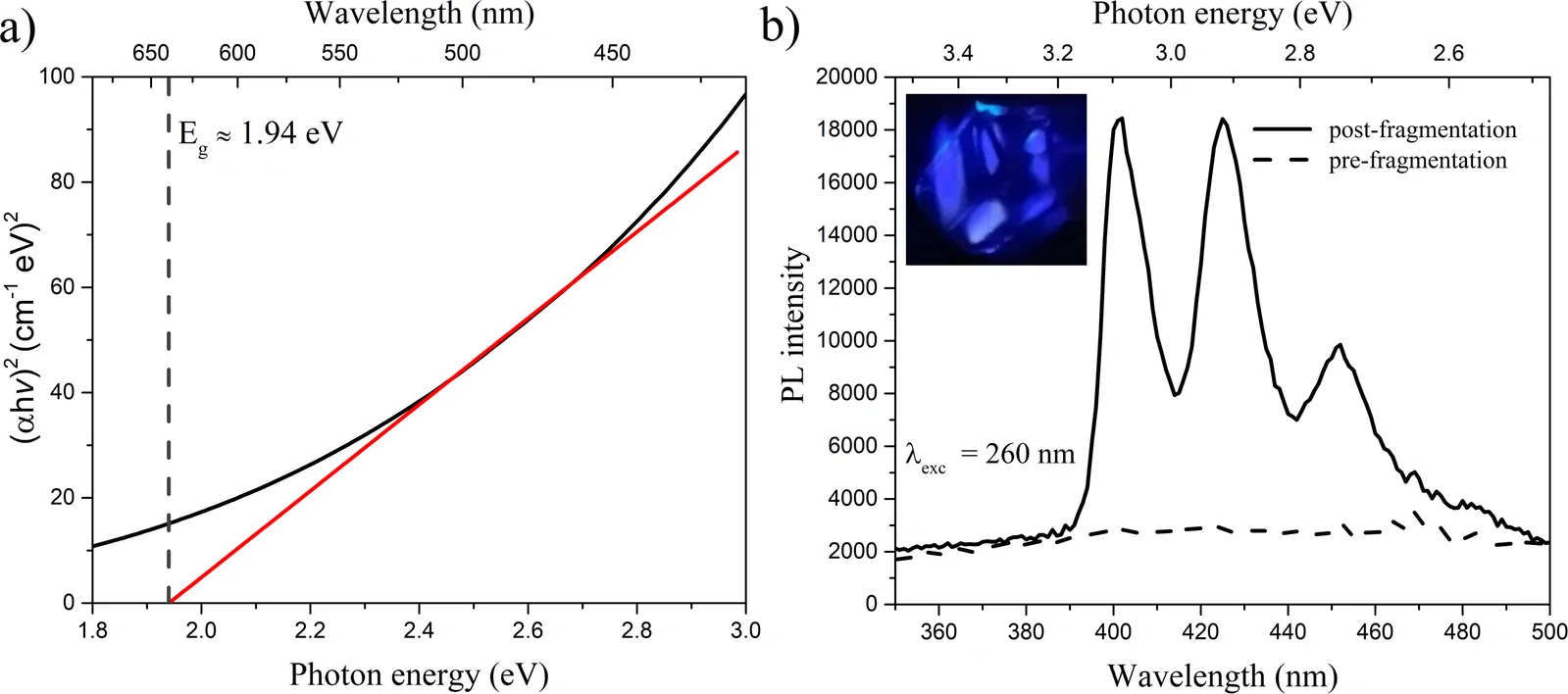
(a) Tauc plot of (α*h*ν)^2^ as a function of photon energy (*h*ν) for the MoS_2_/MoO_3−_*_x_* colloidal suspension obtained from the UV–vis absorbance spectrum. The linear region (red line) was extrapolated to the energy axis, yielding an optical band gap of approximately 1.94 eV. The linear regression was performed over the range 2.183–3.039 eV (81 points), with *R*^2^ = 0.984. (b) PL spectra of the as-ablated (pre-fragmentation) and colloidally fragmented (post-fragmentation) MoS_2_/MoO_3−_*_x_* colloidal solutions under λ_exc_ = 260 nm. The as-ablated colloid remains essentially non-emissive, whereas the post-fragmentation sample exhibits an intense blue–violet emission, confirming the strong photoluminescent response of the fragmented nanocomposite in the visible region.

The optical bandgap (*E*_g_) of the MoS_2_/MoO_3−_*_x_* colloidal solution was estimated from a Tauc plot constructed from the UV–vis absorbance data (Figure 1a), yielding *E*_g_ ≈ 1.94 eV by linear extrapolation. The optical bandgap was estimated using the Tauc relation for an allowed direct transition (*n* = 2), that is, a linear fit of (α*h*ν)^2^ versus *h*ν, where α is the absorption coefficient, *h* is Planck’s constant, and ν is the frequency of the incident photon. The linear regression was performed over the range of 2.183–3.039 eV (81 points), after excluding the exponential tail near the absorption edge (Urbach tail), and gave *R*^2^ = 0.984. Displacing the limits of the fitted region by ±0.1 eV changed the extrapolated value of *E*_g_ by less than 0.08 eV, indicating that the extrapolation is not critically dependent on the choice of fitting window. The Urbach region was excluded because its exponential contribution, commonly associated with disorder/defects, can distort the slope of the absorption edge; if included, it may lead to an under- or overestimation of *E*_g_ [25–26]. The obtained value (1.94 eV), higher than the indirect bandgap of bulk MoS_2_ (~1.2 eV), falls within the range reported for monolayer/few-layer MoS_2_ (~1.8–1.9 eV), where thickness reduction can shift the material from an indirect- to a direct-gap regime [27]. In curved inorganic fullerene-like MoS_2_ (IF-MoS_2_), optical absorption indicates preserved semiconducting behaviour with exciton energies shifting relative to bulk 2H-MoS_2_ as a function of size and layer number. As reported by Frey et al., such shifts can move the optical edge toward values comparable to those observed here (~1.94 eV), in agreement with thin and/or curved domains [28]. Overall, the estimated *E*_g_ is consistent with confined and/or curved MoS_2_ domains, as expected for fullerene-like or nanospherical MoS_2_ nanostructures.

Additionally, theoretical and experimental studies of MoS_2_/MoO_3_ interfaces indicate that the interaction between a two-dimensional transition metal dichalcogenide (TMD) and a transition metal oxide (TMO) can modify band alignment, generate interfacial states, and modulate the effective absorption near the edge [29–30]. In our case, the presence of a sub-stoichiometric MoO_3−_*_x_* shell surrounding a curved/nanometric MoS_2_ core provides a plausible framework to rationalize two features: (i) an apparent optical edge at ~1.94 eV, mainly associated with the thin/curved MoS_2_ component (with a more direct-like character); and (ii) a Tauc slope/curvature that may be slightly influenced by disorder/defect states in the MoO_3−_*_x_* coating (e.g., a more pronounced Urbach tail and possible mixing of transitions) [31].

The photoluminescence (PL) of the MoS_2_/MoO_3−_*_x_* colloids exhibits three main peaks at approximately 401, 425 and 452 nm, corresponding to violet–blue emission (Figure 1b). Notably, this PL response becomes evident after the colloidal fragmentation step, whereas the as-ablated colloid remains essentially non-emissive under the same excitation conditions, as shown by the comparison of the pre- and post-fragmentation spectra in Figure 1b. The first two peaks (~401 and ~425 nm) can be attributed to excitonic transitions in strongly confined MoS_2_ nanodomains. The nanometer-scale size of these domains can broaden the bandgap and shift the emission toward higher energies [5,32–34]. For example, MoS_2_ quantum dots with diameters of only a few nanometers exhibit intense blue fluorescence (typical maximum ~415 nm) under UV excitation [32], which is in line with the appearance of peaks at ~401–425 nm. These emissions are commonly associated with direct electronic transitions in highly confined MoS_2_ regions and may be enhanced by edge defects or surface states in such nanoparticles [5].

In contrast, the third peak (~452 nm) is likely related to defect states in the MoO_3−_*_x_* coating. Oxygen vacancies and reduced centres (Mo^5+^) in sub-stoichiometric molybdenum oxides are known to introduce intragap levels that can act as luminescent centres [35]. Radiative recombination through these defects can produce blue emission; for instance, peaks around 450–465 nm have been reported in MoO_3_ nanofibres with abundant oxygen vacancies [35], which is compatible with the ~452 nm peak observed here. In summary, the ~401/425 nm bands are associated with quantum confinement in MoS_2_ (excitonic transitions in nanostructured domains), while the ~452 nm band is linked to defect-related transitions in MoO_3−_*_x_* (oxygen-vacancy centres) [35]. These assignments provide a basis for future studies exploring structure–defect–emission relationships and application-specific performance.

**Figure 2:**
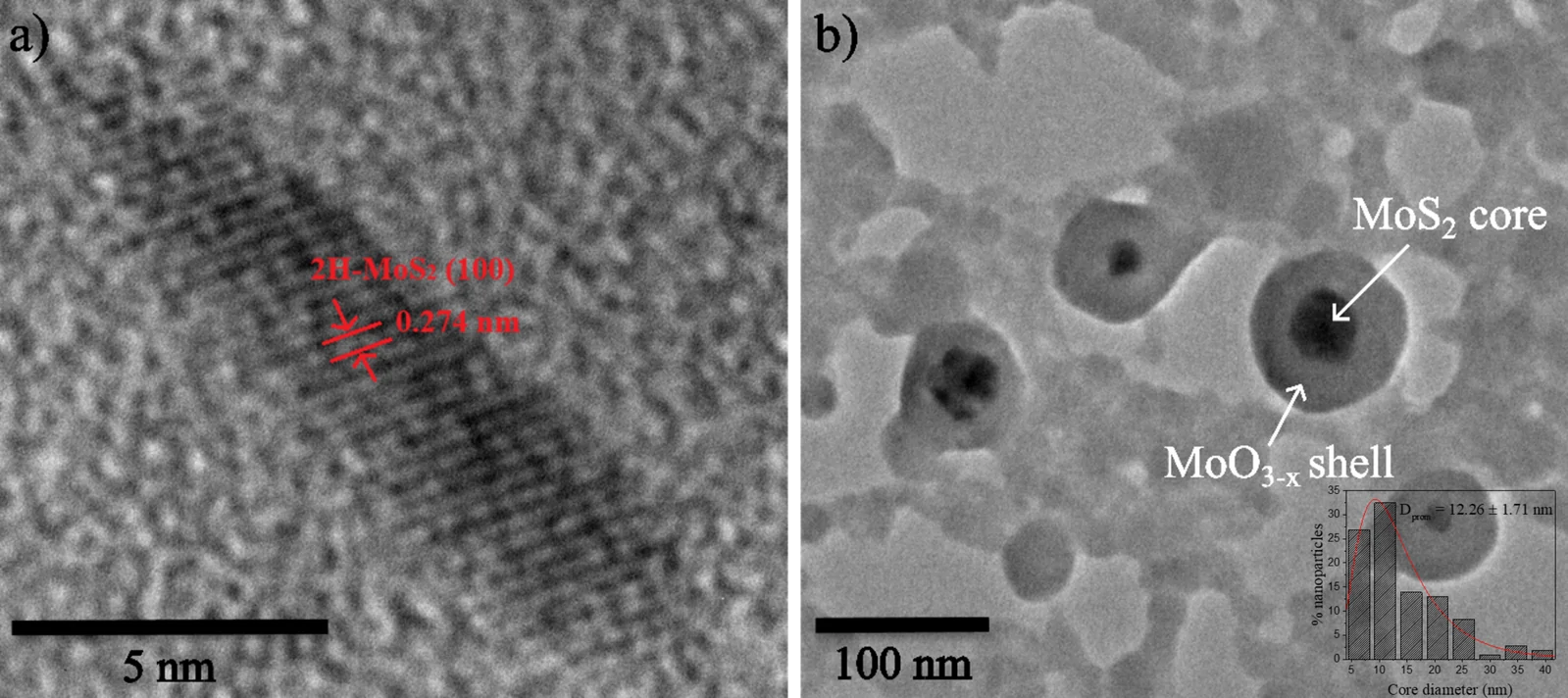
TEM micrographs of the MoS_2_/MoO_3−_*_x_* nanostructures at different stages of the synthesis. (a) High-resolution TEM micrograph of a MoS_2_ nanosheet obtained after the laser ablation step, showing well-defined lattice fringes with an interplanar spacing of ~0.274 nm indexed to the (100) plane of 2H-MoS_2_. (b) TEM micrograph of the sample after the laser-induced fragmentation stage, where spherical fullerene-like core–shell nanostructures are observed, consisting of a darker MoS_2_ core surrounded by a lighter MoO_3−_*_x_* shell. The inset shows the size distribution of the MoS_2_ cores, with an average diameter of 12.26 ± 1.71 nm.

To better understand the origin of these confined domains and defective oxide regions, it is necessary to analyse the structural evolution of the nanomaterials during synthesis. In the first stage, laser ablation of the solid MoS_2_ target in ethanol produces a distribution of MoS_2_-derived nanostructures in the colloidal solution. TEM images acquired before fragmentation (i.e., after ablation) show that nanosheet-like morphologies are predominant at this stage. The HRTEM micrograph in Figure 2a reveals well-defined lattice fringes, and the measured interplanar spacing (~0.274 nm) can be indexed to the (100) plane of hexagonal 2H-MoS_2_. This *d*-spacing agrees well with reported values for the 2H phase [5,36], supporting the assignment of these lamellar structures to 2H-MoS_2_ nanosheets rather than other polymorphs (e.g., 1T), and indicating that the ablation step largely preserves the 2H crystal structure of the initial target. These sheets can be described as exfoliated/lamellar fragments or 2D particles generated during ablation. Previous studies have shown that, under suitable conditions, laser processing of MoS_2_ in liquids can yield spherical “inorganic fullerene”-like nanoparticles (10–15 nm) [37]. In the present case, however, the pre-fragmentation TEM micrographs are dominated by lamellar (2D) structures, suggesting that fullerene-like particles are not yet a major fraction immediately after ablation and that partially ordered sheets and aggregates are formed instead. It is also worth noting that chemical reactions can occur during laser ablation in liquids; for example, reviews of MoS_2_ laser ablation in organic solvents (including ethanol) report the concurrent formation of molybdenum oxides alongside MoS_2_, attributed to reactive species generated at the plasma–liquid interface [38].

In the second stage of the synthesis, the MoS_2_ target was removed and the colloidal suspension containing the 2H-MoS_2_ nanosheets was further irradiated for 60 min with the pulsed Nd:YAG laser. In this post-ablation regime, the laser energy is deposited directly onto the dispersed 2H-MoS_2_ sheets and their aggregates, rather than onto a bulk target. In laser irradiation/processing in liquids, this configuration has been shown to promote additional fragmentation, melting, and reshaping of pre-formed nanostructures in colloidal media [39–41]. Under these conditions, repeated laser excitation, transient plasma formation, cavitation bubbles, shock waves, and local heating around the nanosheets can facilitate edge reconstruction and bending of lamellae. Accordingly, the initially flat 2D nanosheets can curve, fold, and close to reduce surface and edge energy. This behaviour is consistent with the formation of quasi-spherical, inorganic fullerene-like MoS_2_ nanoparticles, as observed in the HRTEM micrographs in Figure 2b after the 60 min irradiation/fragmentation step. Similar laser-driven transformations of lamellar MoS_2_ precursors into fullerene-like nanospheres have been reported for MoS_2_ processed in liquid environments. For example, 10–15 nm IF-MoS_2_ particles have been obtained via laser processing in liquids, with the transformation attributed to laser-induced exfoliation, defect creation, and curvature-driven reconstruction of the layered dichalcogenide [37]. This curvature-driven evolution from planar sheets to closed shells is also in line with thermodynamic arguments and earlier work on IF-MoS_2_ and related layered chalcogenides, which highlight the role of bending strain, dangling-bond reduction, and layer closure in stabilizing hollow fullerene-like morphologies [13].

In parallel with this morphological transformation, prolonged irradiation of the colloid in ethanol can also favour partial oxidation and chemical modification of the newly formed MoS_2_ fullerene-like nanoparticles [42]. Nd:YAG irradiation of alcohols has been reported to induce liquid breakdown under certain conditions, producing transient plasma channels, microbubbles and shock waves, and generating reactive species through solvent dissociation [43]. In our picosecond regime, solvent-derived reactive species (together with dissolved O_2_) may interact with the highly curved MoS_2_ surface, particularly at defect sites introduced during bending and closure, thereby promoting gradual formation of an oxygen-deficient MoO_3−_*_x_* layer at the particle exterior. Laser-driven fragmentation of MoS_2_ platelets in liquids has been shown to yield MoS*_x_* nanocrystals with tunable composition, where oxidation and non-stoichiometry evolve with irradiation conditions [44]. Likewise, liquid-phase laser processing can produce MoS_2_-based core–shell nanostructures in which a more defective or compositionally distinct region surrounds a crystalline MoS_2_ core [45]. The core–shell assignment proposed here is based on the combined evidence of the HRTEM contrast and lattice-fringe distribution, the Raman signatures of both phases, and the optical response of the colloid. In line with this picture, HRTEM micrographs after 60 min irradiation show quasi-spherical particles with a darker interior and a lower-contrast outer region, consistent with a core–shell morphology where 2H-MoS_2_ fullerene-like particles are partially coated by a defective MoO_3−_*_x_* shell formed in situ during laser processing in ethanol. Within the same micrograph, the relative extent of the two regions varies from particle to particle, including among particles of comparable overall diameter, which is not expected from projection contrast alone; such variability is anticipated for laser fragmentation in liquid, where individual particles follow different cumulative irradiation histories, so that the extent of surface conversion differs between particles. The formation of this oxide layer is also apparent macroscopically: The as-ablated suspension is grey, whereas the fragmented colloid is yellow, in agreement with the formation of molybdenum oxide reported for laser processing of molybdenum in oxygen-containing liquids [46].

**Figure 3:**
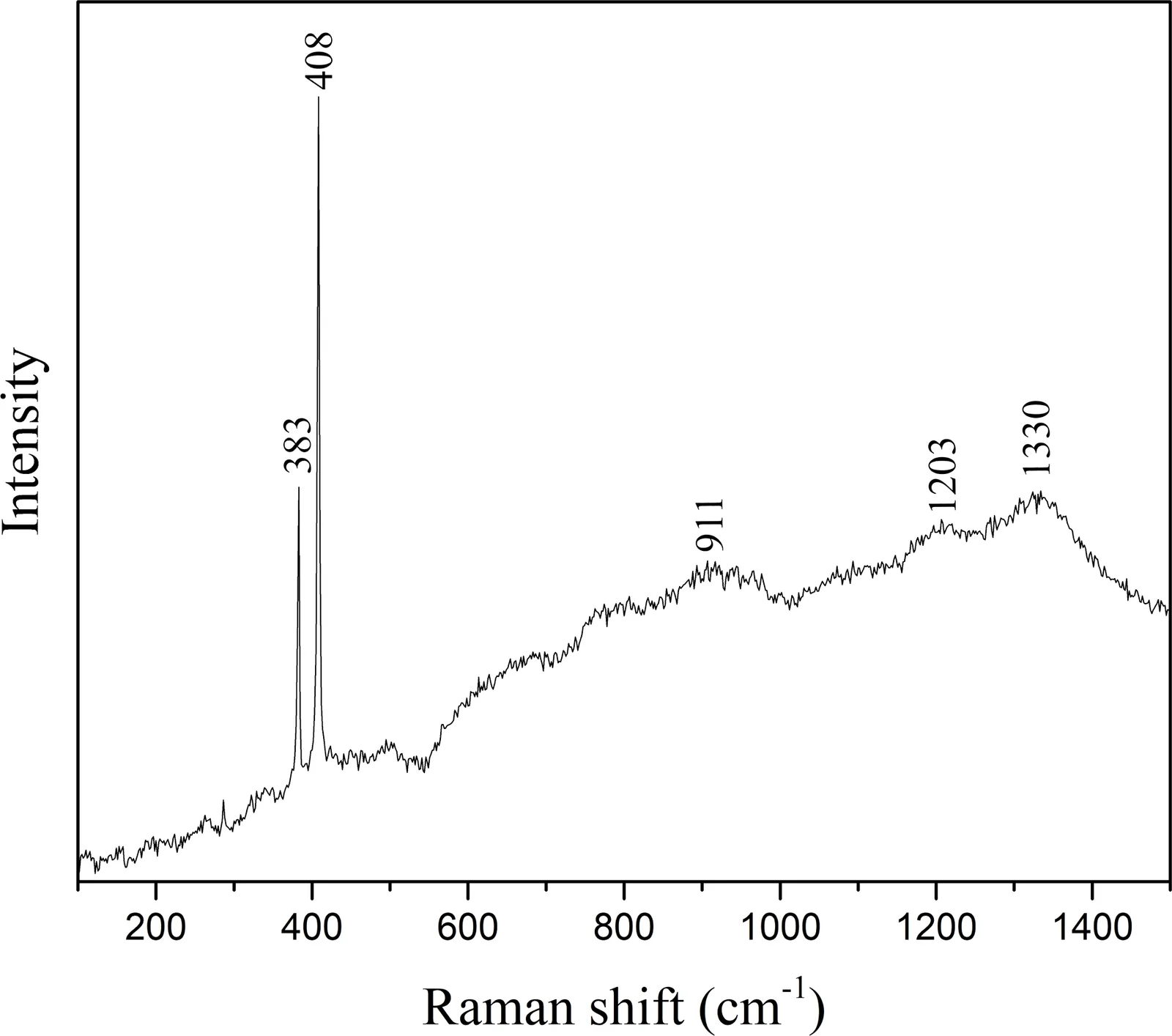
Raman spectrum of the MoS_2_/MoO_3−_*_x_* core–shell nanostructures. The two intense peaks are assigned to the characteristic vibrational modes of the 2H-MoS_2_ core, confirming the presence of MoS_2_ at the centre of the nanoparticles. In addition to these modes, the broad ascending background/band underlying the spectrum is associated with the sub-stoichiometric molybdenum oxide (MoO_3−_*_x_*) that forms the outer shell, whose defective structure contributes to the extended Raman signal.

This nanoscale state is further supported by the Raman spectra shown in Figure 3. The two intense peaks at ~383 and ~408 cm^−1^ are assigned to the 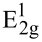 mode (in-plane Mo–S vibration) and the A_1g_ mode (out-of-plane vibration of S atoms) of the 2H-MoS_2_ phase, respectively. These positions fall within the range commonly reported for few-layer and multilayer 2H-MoS_2_, where these modes typically appear at 382–385 and 407–410 cm^−1^ [47]. The separation of ~25 cm^−1^ is characteristic of multilayer-like stacking rather than a monolayer limit, indicating that the processed material retains 2H-type stacked domains. In fullerene-like MoS_2_ nanostructures, these modes can remain near their typical positions but may broaden due to curvature, confinement, and defects [28]. Accordingly, small variations in peak position and/or linewidth are expected for curved MoS_2_ domains. Previous studies have shown that curvature and reduced crystallite size can induce slight frequency shifts and broadening of MoS_2_ Raman peaks [48]; for example, shifts in the 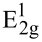 and A_1g_ modes have been reported for MoS_2_ nanotubes/fullerenes and attributed to curvature-induced strain. In addition, defects or edges in nanometer-sized MoS_2_ can activate normally weak modes, such as a band near 226–227 cm^−1^ (LA(M)). In our sample, a distinct peak at ~226 cm^−1^ is not clearly resolved; however, this mode has been reported to appear weakly in MoS_2_ nanomaterials with high edge/defect density [48]. Overall, the peaks at ~383 and ~408 cm^−1^ confirm the presence of 2H-MoS_2_ domains in the nanostructures, while subtle changes in position or linewidth are compatible with size, confinement, and curvature effects.

Above 600 cm^−1^, there is a very broad ascending shoulder overlaid by weak bands at 911, 1203, and 1330 cm^−1^. The signal around 900–950 cm^−1^ is consistent with the stretching modes of Mo=O and Mo–O–Mo in sub-stoichiometric molybdenum oxides (MoO_3−_*_x_*). In the presence of oxygen vacancies, intense bands have been reported, as well as broadening and shifting of the high-frequency bands. Furthermore, studies on MoO_3−_*_x_* show that the concentration of oxygen vacancies correlates with resonant modes in the 800–1000 cm^−1^ region and with IVCT-type electronic transitions around 2 eV [49]. These transitions can generate an increasing Raman background towards higher shifts, as seen in the spectrum. In molybdenum oxide materials that are not fully stoichiometric, it is common to observe these broad Raman bands in the region of 800–1000 cm^−1^ due to vibrations of the Mo=O bonds affected by defects. For example, amorphous MoO*_x_* films or clusters show very broad peaks at 850–950 cm^−1^ instead of the sharp lines of crystalline MoO_3_; this amorphous MoO*_x_* sample exhibited two broad, weak bands at 879 and 952 cm^−1^, attributed to sub-stoichiometric polycrystalline agglomerates of MoO*_x_* rather than a defined crystalline phase [49]. This is very similar to what we see at 911 cm^−1^ in our spectrum: A broad band at 900 cm^−1^ suggests the Mo=O terminal vibration modified by oxygen vacancies. Oxygen vacancies (Mo in oxidation states below +6) weaken the Mo=O bond and tend to lower its stretching frequency: Instead of at ~995 cm^−1^ (Mo=O in pure Mo^6+^), it appears around 900–950 cm^−1^ when there is Mo^5+^ and disorder [49–50]. In fact, in MoO_3_ nanowires with defects, a broad band at ~1004 cm^−1^ was observed, specifically associated with oxygen vacancies (Mo^5+^ centres) and modes activated by relaxation of selection rules [50]. In our case, the band centred at 911 cm^−1^ could indicate Mo=O terminal stretches in defective environments (perhaps MoO_4_ tetrahedra with some absence of bridging oxygen) typical of a partially reduced oxide.

The bands at ~1203 and ~1330 cm^−1^ are not characteristic of pure MoS_2_, but they coincide with regions where carbon defect modes (D_4_ bands at ~1200 and ~1350 cm^−1^) typically appear in disordered carbonaceous materials [51]. Since the synthesis is carried out in ethanol exposed to laser ablation, it is reasonable to attribute these signals, at least in part, to carbonaceous species or surface amorphization caused by solvent decomposition [49]. This could be mixed with higher-order Mo–O modes and Mo–O phonon combinations [49]. All of these results support the idea that the MoO_3−_*_x_* layer coating the fullerenes is sub-stoichiometric and rich in defects.

After establishing the chemical, structural, and optical characteristics of the MoS_2_/MoO_3−_*_x_* nanocomposite, its photothermal response under near-infrared (NIR) irradiation was subsequently evaluated. The MoS_2_/MoO_3−_*_x_* system exhibited a thermal jump of approximately 11 °C under NIR irradiation (270 W for 10 min in 1.5 mL of solution), which is nearly double the thermal jump observed in pure ethanol (approximately 6 °C). It is worth noting that the optical attenuation of a colloid is the sum of absorption and scattering, and that only the absorbed fraction is converted into heat, since scattered photons are redirected rather than deposited in the medium [52]. For particles of the size measured here (~12 nm) the size parameter lies well within the Rayleigh regime, in which scattering scales as λ^−4^. The measured extinction of the colloid, however, increases by only a factor of 2.2 between 800 and 400 nm, compared with the factor of 16 expected for Rayleigh scattering over the same interval. This, together with the yellow colour of the fragmented colloid noted above, indicates that the attenuation is dominated by wavelength-selective absorption rather than by scattering.

**Figure 4:**
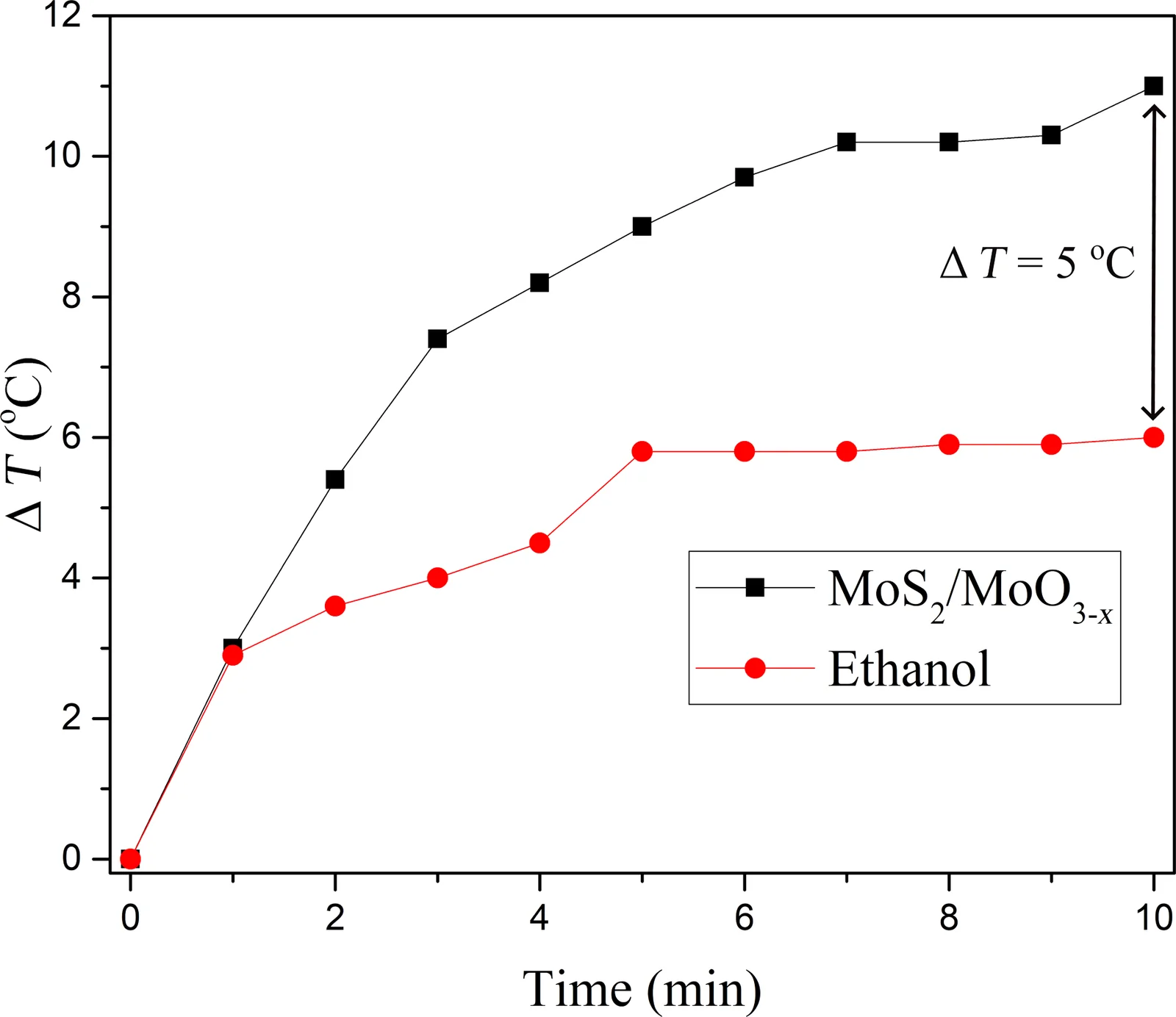
Photothermal heating curves of the MoS_2_/MoO_3−_*_x_* colloidal suspension and ethanol under continuous irradiation with a 270 W infrared lamp. The plot shows the evolution of temperature as a function of irradiation time (0–10 min), where the hottest-spot temperature in each sample was recorded every minute using a thermal imaging multimeter. The MoS_2_/MoO_3−_*_x_* (black squares) exhibits a progressive temperature increase that reaches a slightly higher plateau than the ethanol control (red circles), indicating a modest but measurable photothermal contribution from the MoS_2_/MoO_3−_*_x_* nanosystem.

The time-resolved thermal profile under NIR excitation is presented in Figure 4. The temperature increase indicates intermediate photothermal performance. For comparison, gold nanorods (AuNRs) increase the temperature by approximately 20 °C [53], copper sulfide (CuS) reaches about 15 °C [54], and bare MoS_2_ reaches only about 10 °C [55]. The better performance in comparison with bare MoS_2_ is attributed to the intrinsic properties of the material: The MoS_2_/MoO_3−_*_x_* heterointerface forms a type-II junction that facilitates spatial separation of photoinduced carriers and promotes efficient nonradiative recombination pathways, thereby enhancing heat generation under NIR excitation [56–57]. In addition, the defect-rich MoO_3−_*_x_* shell introduces mid-gap states and broadens the absorption profile, enabling more effective light harvesting and improved phonon coupling compared with bare MoS_2_ [58–59]. Taken together, these factors rationalise the higher Δ*T* observed for this system relative to pure ethanol and bare MoS_2_, rendering it competitive with other reported photothermal platforms. More significantly, the response reported here emerges entirely from the processing: Starting from a colloid that is essentially non-emissive after ablation, a single laser fragmentation step, without any added reagent, produces particles that combine visible photoluminescence with an enhanced NIR heating response. Both properties are therefore accessible through physical parameters alone (pulse duration, fluence, and irradiation time), which illustrates the potential of picosecond laser processing in liquids to modulate the optical and photothermal response of a transition metal dichalcogenide without recourse to capping agents, noble metals, or chemical reagents.

From an application standpoint, NIR-induced temperature increments are often discussed in the context of photothermal therapy, where the key requirement is reaching a target absolute temperature (e.g., mild hyperthermia around 41–45 °C) under defined irradiation and heat-dissipation conditions [60–62]. In the literature, reported temperature rises vary widely (often in the 10–20 °C range in bench-top assays), depending on experimental parameters such as nanoparticle concentration, optical density at the excitation wavelength, irradiation power density, sample volume, and thermal losses [61–67]. While the present study evaluates heating only in colloidal solution using a commercial NIR lamp and does not include biological validation, the observed heating enhancement (~5 °C relative to ethanol under our test conditions) demonstrates a measurable NIR-lamp heating contribution of the MoS_2_/MoO_3−_*_x_* colloids. Together with their UV-excited photoluminescence, these materials provide a materials-level basis for future optimization and application-specific testing under biologically relevant conditions.

The heating reported here is obtained under broadband, spatially distributed irradiation rather than under a focused laser beam. This configuration was chosen deliberately: Focused laser exposure concentrates the incident power on a thin surface layer, which is a recognised source of pain and tissue damage and a known constraint on the radiation levels that can be applied in photothermal applications [8]. That a measurable temperature rise is obtained under distributed irradiation, using a low-cost and readily reproducible lamp arrangement, is therefore of interest in this context. Within this configuration, the response is expected to improve with the density of oxygen vacancies, which can be tuned through the fluence and duration of the fragmentation step [46,68], as well as with colloid concentration and optical density at the irradiation wavelength.

## Conclusion

In this work, we show that picosecond laser ablation in liquids followed by laser-induced colloidal fragmentation provides a clean and versatile route to surfactant-free MoS_2_/MoO_3−_*_x_* fullerene-like nanostructures with visible photoluminescence and a measurable NIR-lamp heating response. Ablation of a high-purity MoS_2_ target in ethanol initially yields predominantly 2H-MoS_2_ nanosheet-like domains with an interplanar spacing of 0.274 nm indexed to the (100) plane, which upon continued irradiation/fragmentation evolve into core–shell fullerene-like nanoparticles with an average diameter of 12.3 ± 1.7 nm. UV–vis absorption and Tauc analysis (direct transition, *n* = 2) give an apparent optical bandgap of *E*_g_ ≈ 1.94 eV, compatible with size/curvature-related modifications in confined MoS_2_ domains. Under 260 nm excitation, the post-fragmentation colloids exhibit intense blue–violet photoluminescence with bands at 401, 425, and 452 nm, interpreted as contributions from confined/curved MoS_2_ regions together with defect-related states associated with oxygen-deficient MoO_3−_*_x_* in the shell. Raman spectroscopy further supports the coexistence of 2H-MoS_2_ features and signatures consistent with sub-stoichiometric molybdenum oxide, in line with the proposed core–shell heterostructure. Photothermal tests performed on the post-fragmentation nanocolloid using a commercial 270 W NIR lamp (1.5 mL, 10 min) show a heating enhancement of ~5 °C relative to ethanol under the same conditions. Overall, the study establishes a laser-processing pathway and links HRTEM- and Raman-based structural/defect signatures with UV–vis/Tauc optical absorption and UV-excited photoluminescence in MoS_2_/MoO_3−_*_x_* fullerene-like nanocolloids. It also provides an initial assessment of NIR-lamp heating in the post-fragmentation nanocolloid. Future work will investigate cellular interactions and photothermal effects in cancer cell lines under controlled irradiation conditions, alongside optical readout via photoluminescence.

## Experimental

For the synthesis, a solid and highly pure (99.9%) molybdenum disulfide (MoS_2_) target (ACI ALLOYS INC) was ablated for 30 min using a Nd:YAG laser (EKSPLA, 1064 nm) with 30 ps pulses, frequency of 10 Hz and a fluence of 0.8 J·cm^−2^, employing 30 mL of reagent-grade ethanol as the liquid medium contained in a 150 mL glass beaker (PYREX). Subsequently, the system underwent a laser fragmentation process for 60 min under the same laser parameters, in which the target was removed, and the pulses were incident directly on the colloidal suspension obtained.

The structural, morphological, and optical characterization of the samples was carried out using high-resolution transmission electron microscopy (HRTEM) (JEOL JEM-2010, 200 kV) to determine morphology, particle size, lattice fringes, and the presence of closed fullerene-like structures; Raman spectroscopy (HORIBA LABRAM HR800, excitation wavelength of 532 nm, spectral range of 100–1500 cm^−1^) to identify crystalline phases and assess surface oxidation effects; UV–vis spectroscopy (PerkinElmer Lambda 650, spectral range of 200–900 nm) to analyse electronic transitions and estimate the optical bandgap; and photoluminescence (PL) spectroscopy (HORIBA FLUOROMAX PLUS, λ_exc_ = 260 nm) to evaluate optical responses associated with structural defects and surface states induced by the ablation and fragmentation processes.

The photothermal heating experiments were performed by irradiating 1.5 mL of the colloidal solution obtained after ablation and fragmentation, as well as 1.5 mL of pure ethanol as a control, each contained in a standard 1.5 mL Eppendorf tube, with a 270 W infrared lamp (VOLTECK) positioned at a fixed distance of 30 cm. During irradiation, the tubes were held horizontally and directly exposed to the lamp without additional stirring or external cooling. The temperature increase of the solutions was monitored in real time using a thermal imaging multimeter (ZOYI) aimed at the central region of each tube. This instrument continuously provided the maximum temperature value within the thermal image, and this hottest-spot temperature was recorded every minute over a total irradiation time of 10 min to construct the corresponding heating curves.

## Data Availability

Data generated and analyzed during this study is available from the corresponding author upon reasonable request.
